# A dose response model for quantifying the infection risk of antibiotic-resistant bacteria

**DOI:** 10.1038/s41598-019-52947-3

**Published:** 2019-11-19

**Authors:** Srikiran Chandrasekaran, Sunny C. Jiang

**Affiliations:** 1 University of California Irvine, Civil and Environmental Engineering, Irvine, 92697 United States; 2 University of California Irvine, Center for Complex Biological Sciences, Irvine, 92697 United States

**Keywords:** Environmental microbiology, Environmental sciences

## Abstract

Quantifying the human health risk of microbial infection helps inform regulatory policies concerning pathogens, and the associated public health measures. Estimating the infection risk requires knowledge of the probability of a person being infected by a given quantity of pathogens, and this relationship is modeled using pathogen specific dose response models (DRMs). However, risk quantification for antibiotic-resistant bacteria (ARB) has been hindered by the absence of suitable DRMs for ARB. A new approach to DRMs is introduced to capture ARB and antibiotic-susceptible bacteria (ASB) dynamics as a stochastic simple death (SD) process. By bridging SD with data from bench experiments, we demonstrate methods to (1) account for the effect of antibiotic concentrations and horizontal gene transfer on risk; (2) compute total risk for samples containing multiple bacterial types (e.g., ASB, ARB); and (3) predict if illness is treatable with antibiotics. We present a case study of exposure to a mixed population of Gentamicin-susceptible and resistant *Escherichia coli* and predict the health outcomes for varying Gentamicin concentrations. Thus, this research establishes a new framework to quantify the risk posed by ARB and antibiotics.

## Introduction

The rise of antibiotic resistance in bacteria coupled with the slowdown of drug discovery presents a growing threat to public health^1^. The Centre for Disease Control and Prevention (CDC) estimates at least 2 million illnesses and 23,000 deaths a year in the US can be attributed to antimicrobial resistance. The economic burden in the US is estimated to be on the order of US $21 to $34 billion^2^.

Quantifying the human health risk associated with antibiotic resistance presents several challenges^3–9^. The human health outcome (e.g., illness not responding to a specific antibiotic or’resistant’ illness) can be influenced by antibiotic resistant bacteria (ARB), antibiotics (ABs), antibiotic resistant genes (ARG) and their carriers. ARB cause antibiotic-resistant illness and present the most direct threat. Antibiotics and other selective pressures (e.g., heavy metals) in the environment promote enrichment of ARB and induce *de-novo* resistance mutations in antibiotic susceptible bacteria (ASB) or the uptake of ARG which is known as horizontal gene transfer (HGT). The ARG can come from direct contact with bacteria harboring ARG (conjugation), from phages harboring ARG (transduction) or from free floating mobile genetic elements (MGEs) in the environment (transformation). HGT can potentially be modulated by the presence of ABs in the environment. Thus the human health outcome from ingesting ARB can be influenced by a complex network of factors.

ARB have been isolated in various sources across the globe including wastewater treatment plant effluent, recreational water, drinking water (see^8^ for a list) and even lettuce at harvest^10^. Yet, microbial risk assessments involving ARB are rare^11,12^, which is due to the lack of a dose response model (DRM) and uncertainties at each step in a traditional bottom-up risk assessment approach. Few past studies (e.g.^13,14^) had investigated the burden of ARB using a top-down approach, in which the contribution of veterinary AB use to the overall number of AB resistant disease instances was investigated^12^. However, this top-down framework cannot be used to compute the risk posed by an exposure event (such as swimming in the recreational waters discussed in^15^), nor can it be used to set regulatory guidelines for acceptable levels of ARB or residual ABs in the environment.

Attempts to relate ARB concentrations to ARB caused diseases (bottom up approach) were made in several studies^16–19^. Since DRMs tailored to ARB don’t exist, these studies draw on epidemiological data (e.g. annual illness cases where some AB fails) to predict human health effects. However, there is a large variability of the estimates depending on the scope and geographic region of the investigation. These past studies are useful to draw inferences on the region that the data are based on but may not be applicable to other regions e.g. resource limited countries where epidemiological data are not available. Moreover, even if data are available, avoiding confounders to pinpoint the true cause of an AB resistant illness is non-trivial. In addition, these approaches are unable to account for the fraction (*f*_*r*_) of ARB in a bacteria contaminated sample or the effects of residual ABs in the environment. Hence, there is a need for DRMs that can account for ARB^8,15^, their fraction (*f*_*r*_) in the dose and residual ABs.

Obtaining data to develop a DRM for human illness involves infecting a cohort of people with a known pathogen, including AB resistant ones. However, since inoculating people with ARB would result in untreatable illnesses, such data are not available. An alternative approach is to use the existing data collected from human studies involving ASB and assume that in the absence of AB, the same DRM used for the ASB is applicable to ARB. This is a conservative assumption since AB resistance often confers a fitness cost^20^. In some cases, mutations that lead to increased fitness have been observed^21,22^. While data from animal models present a second alternative, usage of human data, where available, is preferred.

The development of DRM for ARB is further complicated when the person under consideration is exposed to residual/sub-inhibitory levels of ABs. This can be due to medication for a previous illness, prophylactic use for surgeries, receiving AB-releasing stents, AB residues in food of animal origin or even the environmental sources listed earlier. The source of ARB could also contain AB, causing the host to get exposed to sub-inhibitory levels of AB. In this case, the health outcome (not ill, AB treatable illness or AB untreatable illness) will not only depend on the initial dose (*d*) and fraction of ARB (*f*_*r*_), but also the amount of residual AB. This is because the AB will affect the growth rates of the susceptible and resistant subpopulations differently in a concentration dependent manner. Popular/classical microbial pathogen DRMs, such as the exponential or *β*-Poisson models, are not easily amenable to investigating such changes in growth rates or conversion from ASB to ARB due to mutation or HGT. Hence there is a need for DRMs based on growth processes, which can set the stage for developing a holistic understanding of the dose response of ARB.

**Figure 1:**
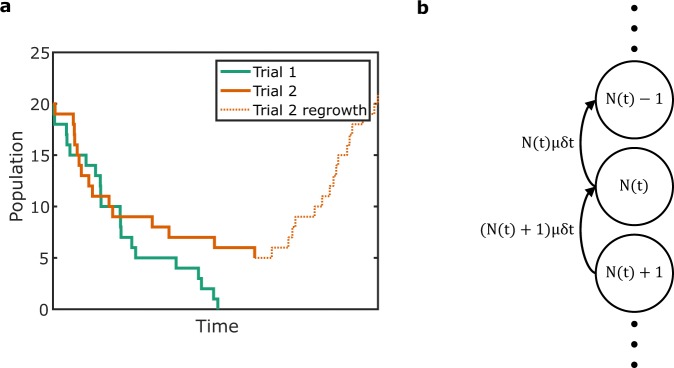
Overview of simple death process. (**a**) Plots of two trials of a simple death process. (**b**) The Markov chain of a simple death process.

The goal of this research is to quantify the risk posed by ARB and the effect of selection pressure exerted by sub-inhibitory concentrations of AB. Specifically, we introduce a new DRM based on Simple Death processes. This DRM begins with the following assumptions: (1) a portion of ingested dose of *d* bacteria may die off (solid lines, Fig. 1a) when they encounter the host’s defenses. This includes immune factors, gastric acids and other factors. (2) At greater *d*, this death rate may not be enough to kill-off all the bacteria. In some cases, enough bacteria survive to initiate an infection (Trial 2, Fig. 1a), resulting in growth (dotted line, Fig. 1a) of bacteria in human body (infection). These two assumptions are modeled by continuous time Markov chains (CTMCs) to capture stochastic bacterial kinetics (Fig. 1b). This differs from existing approaches where kinetics are derived from classical dose response assumptions^23^ or where deterministic kinetics are used to inform dose response^24,25^. Our approach is similar to the approaches of^26–28^ and other studies that use CTMCs, but uses analytical solutions instead of simulations. Another study^29^ explores an analytical approach specific to anthrax and uses *in vivo* animal data to fit parameters. Yet, it is not clear if parameters found in animals apply to humans. Here, we show that our approach, by integrating with classical DRMs, can be used for all pathogens analyzed with the classical DRM framework.

The manuscript is organized as follows. The new DRM model is introduced and fitted to published dose response data of AB susceptible *E. coli* that causes diarrhea. A relationship between the kinetic constants in the proposed model and the existing DRMs is identified. This relationship is then used to predict the risk of illness and the illness subtype (AB treatable vs AB untreatable) for mixed doses of ARB and ASB in the presence of residual AB. Finally, the limitations of this approach and the need for improved data collection complementing this approach are highlighted. Parameters and their symbols presented in the paper are listed in Table 1.

**Table 1:** Parameters used in this study.

Symbol	Units	Description
*C*	mgL^−1^	AB concentration
*d*	CFU	Dose of bacteria
*E*_max,_ EC_50_	day^−1^, mgL^−1^	AB-bacteria interaction parameters
*f*_*r*_	—	Fraction of ARB in initial dose
*n*_ill_	persons	Number of ill subjects
*n*_tot_	persons	Total number of subjects
*P*(*d*,*t*)	—	Response probability for initial bacterial load of *d* at *t*
*P*_ext_(*d*,*t*)	—	Probability of extinction of initial bacterial load of *d* at *t*
*P*(*d*)	—	Response probability for initial bacterial load of *d*
*r*	CFU^−1^	Exponential DRM parameter
*t*	days	Time
*t*_fs_	days	The latest time at which a first symptom is observed
*α*,*β*	—, CFU	*β*-Poisson DRM parameters
*μ*	day^−1^	Death rate of bacteria
*μ*_*s*,AB_(*C*)	day^−1^	Death rate of ASB in AB of concentration *C*
*μ*_*r*,AB_(*C*)	day^−1^	Death rate of ARB in AB of concentration *C*

## Results

### Dose response expression

The initial die-off of the bacteria after they enter the host can be modeled as a stochastic *death process*, which is a kind of CTMC. In such a process, a single death is assumed to occur at a random point in time (solid lines, Fig. 1a). The key assumption behind *Simple* Death (SD) process is that in the short time interval (*t*, *t* + *δt*), each bacterium dies with probability *μδt*. Hence, the probability of a death in the time interval (*t*, *t* + *δt*) is *N*(*t*)*μδt*, where *N*(*t*) is the population size at time *t*. As time goes by, *N*(*t*) will reduce as some bacteria begin to die, thus decreasing the probability of a death in the time interval (*t*, *t* + *δt*). This process is represented by the CTMC in Fig. 1b. Assuming that the probability of observing a response (here illness) equals the probability of not observing an extinction, the relationship can be expressed as Eq. (1).

1 $$P(d,t)=1-{P}_{{\rm{ext}}}(d,t)$$

where *P*(*d*, *t*) is the probability of observing a response and *P*_ext_(*d*, *t*) is the probability of extinction for initial dose *d* and time *t*. The expression for *P*_ext_(*d*, *t*)^30^ is given by

2 $${P}_{{\rm{ext}}}(d,t)={(1-\exp (-\mu t))}^{d}$$

Intuitively, 1 − exp(−*μt*) is the probability of the death of one bacterium^30^. The probability of *d* bacteria dying is the product of the probability of each bacterium dying, resulting in Eq. (2). Therefore, the time dependent dose response relationship or the time dependent SD DRM can be expressed as:

3 $$P(d,t)=1-{(1-\exp (-\mu t))}^{d}$$

*t* in the model (Eq. (3)) is set to the latest time that the first symptom is observed among all subjects (*t*_fs_) in the clinical feeding study. This is because at *t* < *t*_fs_, the *n*_ill_ used to fit the model is higher than the number of people ill at that time. At *t* > *t*_fs_, the die-off assumption will not hold as the bacterial population will enter the growth phase in the human body, which results in illness. Hence the time independent dose response relationship is given by Eq. (4).

4 $$P(d)=1-{(1-\exp (-\mu {t}_{{\rm{f}}s}))}^{d}$$

Here, *d* is the dose of bacteria that is ingested, *P*(*d*) is the response probability, *μ* is the rate of death, and *t*_fs_ is the latest time at which the first symptom is observed among all subjects. We refer to this model as the SD DRM through the remainder of the paper unless specified otherwise.

### Relationship with the existing DRMs

The exponential DRM^31^ is a widely used and best accepted model for dose response of pathogenic *E. coli* in humans. It is expressed as

5 $$P(d)=1-\exp (\,-\,rd)$$

where *r* is the probability of the pathogen surviving to cause infection once it is ingested. When comparing the SD DRM with the exponential DRM, it is clear that exponential DRM is a special case of the time dependent SD DRM (Eq. (3)) where *t* = *t*_fs_.

6 $$(1-\exp (\,-\,\mu {t}_{{\rm{fs}}}))=\exp (\,-\,r)$$

This result implies that SD DRM will fit any dataset that the exponential DRM fits. In addition, the SD DRM establishes a link between an abstract parameter that is informed by dose response data (*r*) and a parameter that has a clear biophysical interpretation (*μ*). This biophysical parameter becomes instrumental in accounting for the effect of the AB.

Similarly, the above approach can also be extended to the existing *β*-Poisson DRM that describes pathogenic *E. coli* dose response, if a relationship between the *β*-Poisson DRM parameters and the death rate (*μ*) of the SD DRM is established. The *β*-Poisson DRM is an approximation of the hypergeometric DRM^31^. The hypergeometric DRM is a generalized case of the exponential DRM, where the *r* value is assumed to be beta distributed. The classical *β*-Poisson is DRM given by:

7 $$P(d)=1-{{\textstyle (}1+{\textstyle (}\frac{d}{\beta }{\textstyle )}{\textstyle )}}^{-\alpha }$$

where *α* and *β* are the parameters of *β*-Poisson model. An analytical approach to finding the required relationship was not tractable, and instead, a numerical approach (see Methods) was successful at finding the solution. With this relationship, the *β*-Poisson model can also be investigated under AB loads. Thus, the results demonstrate SD approach provides a unified framework to analyze the effect of ABs on dose response.

### Accounting for residual AB concentration

In addition to the dose of ARB (or the *f*_*r*_, the fraction of resistant bacteria in the dose), the human health outcome also depends on *C*, the concentration of sub-lethal/residual AB in human body. To account for the effect of AB, we can first adopt models that relate AB concentration and *E. coli* death rate published in literature e.g.^32–34^). These death rates can then be related to dose-response by using Eq. (6).

AB causes a significant increase in the death rate of ASB but has less or no effect on ARB. Hence, a simple conservative assumption is that ARB death rate is not affected by the presence of AB. This assumption is the worst-case scenario because it does not include the fitness cost of AB resistance^20^. This relationship is illustrated in a case study, in which the effect of Gentamicin on a dose of *E. coli* is investigated. The increased death rate of the susceptible strain in the presence of AB (*μ*_*s*,AB_) is given by the sigmoidal model of^33^.

8 $${\mu }_{s,{\rm{AB}}}(C)=\mu +\frac{{E}_{{\rm{\max }}}C}{E{C}_{50}+C}$$

*C* is the concentration of antibiotic. *μ* is the death rate of the ASB strain in the absence of AB. It can be obtained from Eq. (6). *E*_max_ (=1224 day^−1^, the maximum killing rate) and EC_50_ (=9.93 mgL^−1^, AB concentration at half maximum killing rate) are values that determine how *C* affects *μ*_*s*,AB_(*C*). Numerical values for *E*_max_ and EC_50_ were obtained from^33^, where the effect of Gentamicin on *E. coli* death kinetics was studied. The death rate of the ARB in the presence of AB (*μ*_*r*,AB_) is simply *μ* as per the earlier assumption. The probability of illness of doses consisting of purely ASB or purely ARB can thus be estimated by plugging the estimated *μ*_*s*,AB_(*C*) or μ_*r*,AB_ repsctively into Eq. (4) (see Supplementary Methods).

### Accounting for fraction of ARB

We account for *f*_*r*_ by first assuming, like in existing DRMs, that any two bacteria act independently of each other. Scaling this up implies that the susceptible and resistant subpopulations will also act independent of each other. Therefore, the joint probabilities concerning both subpopulations can be written as the product of the probabilities of each subpopulation. For example, the probability of both subpopulations going extinct (*S*_ext_*R*_ext_ in Table 2) is given by *P*_ext,*s*_(*d*|*f*_*r*_, *C*) × *P*_ext,*r*_(*d*|*f*_*r*_, *C*). Therefore the probability of response (*P*(*d*|*f*_*r*_, *C*)) is equal to the complement of the probability of both populations going extinct i.e,

9 $$P(d|{f}_{r},C)=1-{P}_{{\rm{ext}},s}(d|{f}_{r},C){P}_{{\rm{ext}},r}(d|{f}_{r},C)$$

where the extinction probability of the susceptible subpopulation is

10 $${P}_{{\rm{e}}{\rm{x}}{\rm{t}},s}(d|{f}_{r},C)={(1-\exp (-{\mu }_{s,{\rm{A}}{\rm{B}}}(C){t}_{{\rm{f}}{\rm{s}}}))}^{d\times (1-{f}_{r})}$$

**Table 2:** Possible outcomes (S: susceptible, R: resistant, _sur_: survives and _ext_: goes extinct).

	*S*_sur_ (1 − *P*_ext,*s*_(*d*|*f*_*r*_,*C*))	*S*_ext_ (*P*_ext,*s*_(*d*|*f*_*r*_,*C*))
*R*_sur_ (1 − *P*_ext,*r*_(*d*|*f*_*r*_,*C*))	(*S*_sur_*R*_sur_) Illness AB Untreatable	(*S*_ext_*R*_sur_) Illness AB Untreatable
*R*_ext_ (*P*_ext,*r*_(*d*|*f*_*r*_,*C*))	(*S*_sur_*R*_ext_) Illness AB Treatable	(*S*_ext_*R*_ext_) No illness

and the extinction probability of the resistant subpopulation is

11 $${P}_{{\rm{e}}{\rm{x}}{\rm{t}},r}(d|{f}_{r},C)={(1-\exp (-{\mu }_{r,AB}{t}_{fs}))}^{d\times {f}_{r}}$$

### Possible health outcomes

**Figure 2:**
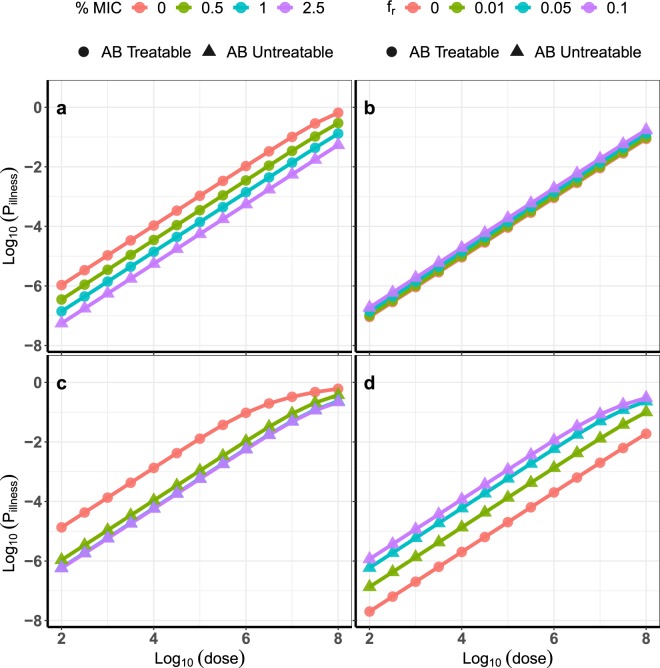
Effect of varying *f*_*r*_ and *C* (as % of MIC: minimal inhibitory concentration) on illness outcomes (AB Treatable; AB Untreatable). (**a**) Exponential DRM, DS1, *f*_*r*_ = 0.05. (**b**) Exponential DRM, DS1, *C* = 1% MIC. (**c**) *β*-Poisson DRM, DS2, *f*_*r*_ = 0.05. (**d**) *β*-Poisson DRM, DS2, *C* = 1% MIC.

Two types of health outcomes are possible when a mixed dose of ARB and ASB are involved. When the ASB strain out-competes the ARB strain (*S*_sur_*R*_ext_, Table 2) to grow and infect the host, the illness would likely be susceptible to the AB treatment under consideration. However, when the ARB subpopulation continues to survive irrespective of what happens to the ASB subpopulation (*S*_sur_*R*_sur_ and *S*_ext_*R*_sur_), the resulting illness may not be treatable with the AB. These health outcomes are labeled as ‘AB Treatable’ and ‘AB Untreatable’ illness, respectively (Fig. 2). To identify the type of health outcome given an illness occurs, we compare the probabilities of these two events and classify as follows:

$${\textstyle \{}\begin{array}{cc}{\rm{A}}{\rm{B}}\,{\rm{T}}{\rm{r}}{\rm{e}}{\rm{a}}{\rm{t}}{\rm{a}}{\rm{b}}{\rm{l}}{\rm{e}}, & (1-{P}_{{\rm{e}}{\rm{x}}{\rm{t}},s}(d,t|{f}_{r},C)){P}_{{\rm{e}}{\rm{x}}{\rm{t}},r}(d,t|{f}_{r},C) > (1-{P}_{{\rm{e}}{\rm{x}}{\rm{t}},r}(d,t|{f}_{r},C))\\ {\rm{A}}{\rm{B}}\,{\rm{U}}{\rm{n}}{\rm{t}}{\rm{r}}{\rm{e}}{\rm{a}}{\rm{t}}{\rm{a}}{\rm{b}}{\rm{l}}{\rm{e}}, & {\rm{o}}{\rm{t}}{\rm{h}}{\rm{e}}{\rm{r}}{\rm{w}}{\rm{i}}{\rm{s}}{\rm{e}}\end{array}$$

Taken together, the risk of infection due to mixed doses (consisting of ARB and ASB) can be estimated as a function of *C*. The host response to AB treatment are predicted based on the subpopulation of the AB resistant *E. coli* ingested. The implementation of this approach differs for the currently used exponential and *β*-Poisson DRMs.

### Effect of ARB fraction ***f***_*r*_ and residual AB concentration ***C***

To understand the effect of ARB fraction *f*_*r*_ and residual AB concentration *C*, the first step is identifying the death rate of the ASB population (*μ* in Eq. (8)). To this end, we fit the existing exponential and *β*-Poisson DRMs to the human clinical datasets (see Supplementary Table S1, data obtained from QMRA Wiki^35^) under consideration. Dataset 1 (DS1) uses mild to severe diarrhea as the endpoint to measure positive response, while dataset 2 (DS2) uses diarrhea. Both datasets use oral as the route of exposure to *E. coli.* The dose and response exhibit a significant trend (at the 0.05 level) for both datasets (one-tailed Cochran-Armitage test^31^, n = 6 and P value = 1.91 × 10^−4^ for DS1, n = 11 and P value = 1.11 × 10^−5^ for DS2).

**Figure 3:**
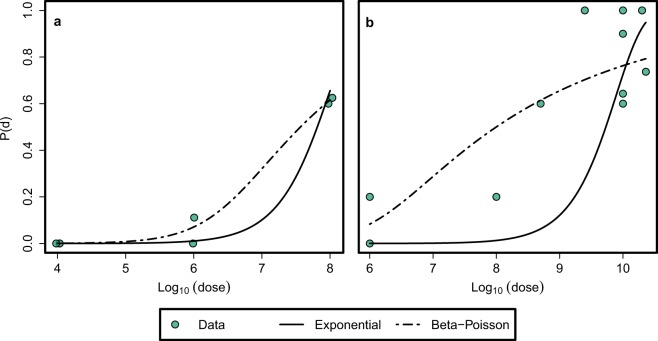
Model fits for (**a**) DS 1 and (**b**) DS 2. To avoid overlapping points in the plot, noise is added along the horizontal axis.

Figure 3 presents the DRM fits and Table 3 summarizes the DRM choice for each dataset based on the *χ*^2^ test, which is used for model selection for DRMs^31^. The results show that the exponential DRM fits DS1 better, while the *β*-Poisson DRM fits DS2 better. The fitted parameters allow us to determine *μ*, which can then be used to estimate the death rate in the presence of AB (*μ*_*s*, AB_) using Eq. (8).

**Table 3:** Fit of the exponential and *β*-Poisson DRMs.

Data	Case	Dev	*χ*^2^	p-value	Conclusion	Model
DS1	Exp	3.19	11.07	0.67	Fail to reject “Exp is a good fit”.	Exp
	*β*-Poisson	0.95	9.49	0.92	Fail to reject “*β*-Poisson is a good fit”.	*r* = 1.07 × 10^−8^
	Compare	2.24*	3.84	0.13	Fail to reject “Exp fits better than *β*-Poisson”.	(*μ* = 18.35 day^−1^)
DS2	Exp	57.82	18.31	9.36 × 10^−8^	Reject “Exp is a good fit”.	*β*-Poisson
	*β*-Poisson	14.44	16.92	0.11	Fail to reject “*β*-Poisson is a good fit”.	*α* = 0.16
	Compare	43.38*	3.84	4.51 × 10^−11^	Reject “Exp fits better than *β*-Poisson”.	*β* = 1.41 × 10^6^

‘Dev’ is the minimum deviance, except the starred (*) values, which are the differences in minimum deviance between exponential and *β*-Poisson DRMs. ‘Model’ represents the preferred DRM based on conclusions. Best fit parameters are also shown.

Figure 2 shows the model behavior at different values of *f*_*r*_ and *C*. For a given dose of bacteria and a given *f*_*r*_, increasing the concentration of AB decreases the risk (Fig. 2a,c). This is expected as the higher AB load kills off more of the bacteria. When *f*_*r*_ = 0.05, increasing *C* from 0 to 2.5% MIC (minimal inhibitory concentration) decreases the risk by around 1.5 orders of magnitude. This decrease in risk is more gradual for DS1 (Fig. 2a) compared to DS2 (Fig. 2c). Not much difference is observed between 1% MIC and 2.5% MIC for DS2, suggesting that the AB effect saturates at small fractions of the MIC. However, increasing *C* also increases the likelihood of the illness not treatable by the AB (Fig. 2a,c, ‘AB Untreatable’). This is attributed to the higher survival capability (or lower death rate) of the ARB subpopulation compared to the ASB subpopulation. Further difference between DS1 and DS2 is observed, as the concentration of Gentamicin that results in predominantly AB untreatable illness is >1% MIC for DS1 but <1% for DS2.

For a given dose of bacteria and a given *C*, increasing *f*_*r*_ also causes an increased risk and greater likelihood of the AB untreatable illness (Fig. 2b,d). The higher risk is due to the lower death rate of the ARB subpopulation. The greater likelihood of AB Untreatable illness stems from the greater number of ARB in the initial load, meaning more ARB are likely to survive with time. Specifically, changing *f*_*r*_ appears to have little effect under DS1 when *C* is fixed to 1% MIC, indicating that AB effect is near saturation. In contrast, increasing *f*_*r*_ from 0 to 0.1 under DS2 increases risk by 2 orders of magnitude, indicating that AB effect reaches saturation at >1% MIC. The switch to predominantly AB untreatble illness occurs at 0.05 < *f*_*r*_ < 0.1 under DS1 but at *f*_*r*_ < 0.01 under DS2.

**Figure 4:**
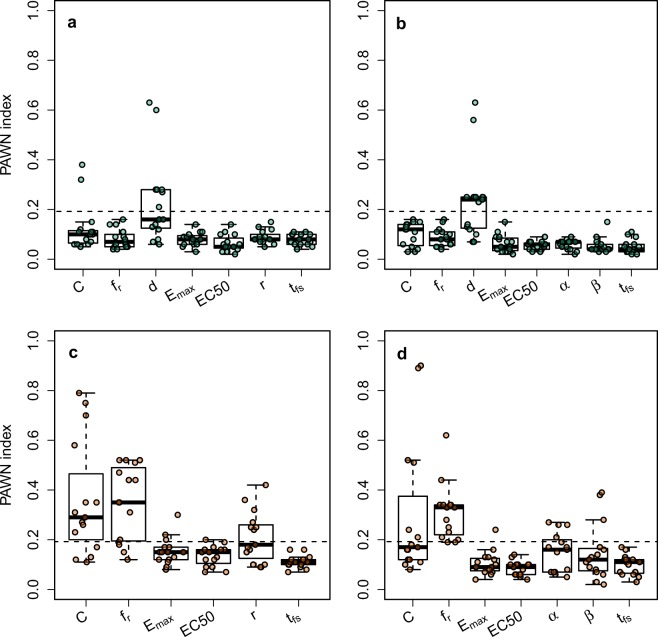
PAWN sensitivity index distributions, higher values are more influential parameters. The dashed line is the threshold value at level of significance = 0.05 (see Methods). (**a**) Exponential DRM, along with dose of bacteria (*d*). (**b**) *β*-Poisson DRM, along with *d*. (**c**) Exponential DRM, fixed *d*. (**d**) *β*-Poisson DRM, fixed *d*.

The magnitude of the impact of *f*_*r*_ and *C* depends on the DRM and the datasets used to fit the DRM (Fig. 2). To better understand this dependence, a sensitivity analysis using the PAWN algorithm^36^ (see Methods) was pursued. The results show that dose of bacteria is the biggest determinant of risk for both the exponential and *β*-Poisson DRMs (Fig. 4a,b). When the dose of bacteria is fixed, *C* and *f*_*r*_ play a bigger role in determining risk than the dose response parameters (Fig. 4c,d). The parameters capturing the effect of the AB (*E*_max_ and EC_50_) play a smaller role than the dose response parameters (Fig. 4c,d). Further, setting *t* = *t*_fs_ is verified to have minimal effect on the final results since sensitivity indices of *t*_fs_ fall below the threshold value, indicating that *t*_fs_ is non-influential (Fig. 4).

## Discussion

While AB resistance is recognized as a growing problem, attempts at developing a quantitative understanding of the risk posed by ARB and the associated sub-lethal AB concentration have been limited. We propose a new approach to modeling the dose response of ARB under the Quantitative Microbial Risk Assessment (QMRA) framework. Under this framework, once a pathogen is identified, human exposure to it is quantified. Exposure is coupled with DRMs to estimate risk of positive response, which is used to design risk management measures.

The crux of the proposed approach is the stochastic process known as the SD process. Kinetics of the bacterial dose is modeled under the SD assumption, resulting in an analytical expression for the extinction probability (*P*_ext_). Like in existing DRMs, response probability is defined as 1 − *P*_ext_. The resulting expression establishes a link between death rate, which is an experimentally observable parameter, and dose response parameters (Eq. (6)). Since the relationship between AB concentration and death rates is known (Eq. (8)), we are also able to relate AB load to dose response. Further, treating the ASB and ARB subpopulations independently, we can compute the odds of the successful treatment with AB in the infected subpopulation.

A major advantage of the proposed approach is that it meshes well with the existing DRMs. An additional choice of DRM, which could complicate the decision of a practitioner, is not necessary. Moreover, the proposed approach does not change how QMRAs are traditionally done. Instead, it provides additional capabilities relevant to AB resistance. It can be used to predict the dose-response of ARB if we have information on (1) the dose-response of ASB; and (2) kinetics of ARB and how it relates to ASB (discussed below). Additionally, if exposure to ABs is suspected in the risk assessment, its influence on human health outcomes can be accounted for using Eq. (8). Since we introduce a novel approach to DRMs, we provide an outline of the steps along with an example (see Supplementary Methods). As shown, the approach works equally well with exponential and *β*-Poisson models to compute dose-response for a single exposure. The total risk from multiple exposures (such as computing annual risk from daily risk estimates) can also be calculated in the usual way by assuming independence between exposures (e.g. one day’s risk doesn’t affect another day’s risk)^31^.

The conservative assumption enabling this approach is that the ARB and ASB subpopulation have the same death rate in the absence of AB. If there is evidence for significant differences in death rates in the absence of AB (due to absence of mutation to compensate fitness loss), then this difference can be set to *μ*′ − *μ*. Here *μ*′ is the ‘base’ death rate for the ARB, which will be higher than the death rate of ASB (*μ*). Then *μ*_*r*,AB_ = *μ*′ + *f*(*C*), where *f*(*C*) is the concentration dependent effect of the AB on the death rate. Another assumption made is that there are only two kinds of bacteria, ARB and ASB. However, resistance may vary in degree in different subpopulations. If and when information (e.g. different *E*_max_ values) on the multiple subpopulations become available, the probabilistic framework can be easily adopted by assuming that each subpopulation acts independently. In fact, the framework presented here can also be used for samples containing a mixture of different pathogens present in an environmental sample, to estimate the total risk due to all the pathogens.

As a proof of concept, we investigate the case study of *E. coli* and the AB Gentamicin. The AB concentration *C* and the fraction of ARB *f*_*r*_ emerge as influential parameters affecting the health outcome from a global sensitivity analysis. A rigorous exposure assessment to quantify *C* and *f*_*r*_ would thus be necessary to get accurate results in a QMRA. The AB-bacteria interaction parameters (*E*_max,_ *EC*_50_) seem less influential for Gentamicin. This supports the use of these parameters, that were determined *in-vitro*^33^, in a host system. A similar analysis would need to be carried out for different AB-bacteria combinations to draw generalized conclusions.

A critique of this approach is that it fails to account for the details in the biological system. For example, the precise concentration of AB in the vicinity of the bacteria is difficult to estimate and likely varies in time. The bacteria themselves translocate from the point of entry to the site of infection (e.g. travel through the alimentary canal). A more detailed stochastic model for dose response (with compartments or spatial distributions) may better reflect the underlying system but would be significantly harder to parameterize effectively. Analytical expressions will also be harder to come by and the approach may require dynamic simulations for dose response. Hence, we believe that the proposed approach provides a framework as a first step to solve the problem. For example, the range of variation in the outcome due to the change in *C* with time can be captured by investigating the outcomes at several fixed concentrations.

When assuming that the probability of observing a response (illness) equals the probability of not observing an extinction (Eq. (1)), we are not accounting for the carrier population. These are the individuals who harbor the pathogen but do not show any visible symptoms (infected but not ill). Insight into the probability of infection in addition to the probability of illness can be obtained if the dataset had information on the number infected. The proposed approach can be used to compute the probability of observing the response (infection). The probability of illness can then be computed by multiplying this quantity by the constant probability of illness given infection (as is done for norovirus in^37^). However, if evidence suggests that ARB are more virulent than the ASB, this approach will underestimate the illness risk. Here we distinguish between virulence (observed *in-vivo*) and fitness or growth rate, which can be observed *in-vitro*. The latter can be accounted for using the death rates, as discussed above. In summary, although the approach discussed here does not explicitly deal with illness and infection, the framework presented can be applied to this end if the data are available.

HGT and spontaneous mutation can result in the creation of ARB, which can potentially influence the type of health outcome. The SD DRM presented here can be modified to investigate the importance of these processes. For example, conjugation is modeled as a second order reaction (first order in ASB population (*d*(1 − *f*_*r*_)) and first order in ARB population (*df*_*r*_))^38^. A conservative estimate of rate constant of conjugation for *E. coli* is *r*_conj_ = 2.4 × 10^−11^ mLcells^−1^ day^−1^ ^38^. Hence the observed rate of conjugation becomes comparable to observed net death rates (*μ* = 18.35 day^−1^ for DS1, Table 3) only if either the ARB or ASB subpopulation number around 10^10^ cellsmL^−1^. This is extremely high and atypical of what is expected from environmental exposure, suggesting that conjugation does not affect dose response significantly. Judging the importance of transduction and transformation is non-trivial as the numbers of phages and MGEs must be accounted for respectively. Yet, if these quantities were known along with the associated rates, the SD framework can be modified to account for them.

The SD framework can be applied to both the exponential and the *β*-Poisson DRMs. Hence, this framework can be used to understand the effect of ABs on other bacteria for which these models are applicable. Yet, for a given organism, the best DRM relies on the objective of the QMRA being pursued and the dataset chosen^35^. In this study, the two different datasets for *E. coli* yield two different best fit models. Risk predictions also depend to a big extent on the dataset chosen. Deciding on the “best” dataset or DRM for *E. coli* is beyond the scope of this paper and is dicussed in the QMRA Wiki website^35^. Nevertheless, our framework accommodates both DRMs and provides the capability to investigate AB resistance.

One limitation of applying this approach is the paucity of experimental data at each step. While *in-vitro* experiments^33^ are used to capture the effect of the concentration of AB on bacteria, most studies on AB-bacteria interactions do not model the concentration-rate relationship, and instead report summary metrics of therapeutic importance^32^. While we have worked only with human datasets in this study, animal experiments may provide better quality data. Yet, whether quantitative conclusions can transfer from animals to humans is debatable, as even human datasets (such as DS1 and DS2) show variability. Another area with insufficient data are the enumeration of ARB. Several studies reporting ARB occurrence in the environment report binary results (presence/absence) for each sample. Others report occurrence summaries such as ‘23% samples tested positive for ARB’ (e.g.^39^). Only few studies (e.g.^40,41^) report the fraction of resistant bacteria in a single sample. Additional data collection at these stages will enable risk assessment case studies.

## Methods

### Fitting the model to data

The model was fitted to two *E.coli* datasets which are listed in Supplementary Table S1 along with the corresponding *t*_fs_. A binomial likelihood was placed on the data as follows:

12 $${n}_{{\rm{ill}}} \sim {\rm{Binomial}}({n}_{{\rm{tot}}},P(d,{t}_{{\rm{fs}}}))$$

This approach is commonly used in building DRMs and amounts to minimizing the deviance presented in^31^. The differential evolution algorithm^42^ from DEoptim package^43^ in R^44^ programming language was used to fit the data. The effect of varying ARB *f*_*r*_ at constant AB concentration *C* = 1% MIC (= 2 *μg*/*mL*) and varying *C* at constant *f*_*r*_ = 0.05 are presented in Fig. 2. The effect of AB concentration *C* is incorporated in the model by increasing the death rate according to Eq. (8).

The analytical approach described above suffices to fit the exponential DRM. However, this DRM fails to provide a satisfactory fit for some datasets, for which the *β*-Poisson DRM provides a better fit and is often used as an alternative. As shown in the case for DS2 (Table 3), the *β*-Poisson DRM fits the data better. However, this model does not have a ready *r* value that can be related to the death rate. A numerical simulation is necessary to parameterize the model to include the individual death rate.

The *β*-Poisson DRM assumes that the survival probability of the pathogen, *r*, follows a Beta distribution (with parameters *α* and *β*). This is different from the exponential DRM where *r* is assumed to be the same for all pathogens. The exact probability of illness can be difficult to compute and the relationship in Eq. (7) is used. To relate *α* and *β* to the death rate of the susceptible strain in the presence of AB (*μ*_*s*,AB_), the following approach is applied:

1. Generate *N* random values of *r* (*r*_1_, *r*_2_, ... *r*_N_) from a Beta distribution with parameters *α* and *β*.
2. Compute the corresponding *μ*_i_ values using Eq. (6) for each *r*_i_.
3. For known values of *C*, *E*_max_, EC_50_, and *μ*_i_, compute ${\mu }_{s,{\rm{AB}},i}$ using Eq. (8).
4. Compute the corresponding *r*_*s*,i_ from ${\mu }_{s,{\rm{AB}},i}$ using Eq. (6).
5. Fit a Beta distribution to the *N* samples of *r*_*s*,i_ and obtain the distribution’s parameters, *α*_*s*_ and *β*_*s*_. These can then be plugged into Eq. (7) to estimate *P*(*d*).

The approach outlined above is straightforward, but in practice, numerical issues are encountered due to the extremely small (≤1 × 10^−16^) values of *r*. Hence the fitdistrplus package^45^ in R was used. This procedure was verified to produce satisfactory estimates (see Supplementary Fig. S1).

### Sensitivity analysis

A global sensitivity analysis was carried out using the PAWN algorithm^36^ (algorithm name formed from author names), which measures sensitivity from the entire probability density of the output rather than just the variance of the output. Sensitivity is not characterized by a point estimate, but a list of estimates (PAWN indices) to give a fuller picture, with higher magnitudes representing more influential parameters. The algorithm parameters are *n* = 15, *N*_u_ = *N*_c_ = 100. Both the exponential and *β*-Poisson DRMs were considered separately. The analysis was re-run after fixing the dose of bacteria to understand the effect of the remaining parameters (Fig. 4). A threshold value was calculated at the 0.05 significance level to identify non-influential parameters (dashed line, Fig. 4). Parameters with PAWN indices entirely below the threshold line are non-influential parameters. The parameter ranges in which sensitivity was investigated are described in Supplementary Tables S2 and S3.

## Supplementary information

Supplementary Information

## Data Availability

Codes reproducing the results in this publication are available on Github at https://github.com/JiangLabUCI/AbResistantDoseResponse.
