# Supplementary material for: A dose response model for quantifying the infection risk of antibiotic-resistant bacteria
Source: Sci Rep. 2019 Nov 19;9:17093. doi: 10.1038/s41598-019-52947-3 (PMC6863845; doi:10.1038/s41598-019-52947-3)
Supplement: Supplementary file 1 — Supplementary Information [file 41598_2019_52947_MOESM1_ESM.pdf]

A dose response model for quantifying the infection risk of antibiotic-resistant bacteria

Srikiran Chandrasekaran[1] Sunny C. Jiang[1,*]

[1]University of California Irvine, Civil and Environmental Engineering, Irvine, 92697, United States
*Corresponding author (sjiang@uci.edu)

Supplementary Materials

Datasets used in this study

ID	[Ref]	Dose	n_{ill}	n_{tot}	t_{fs}(days)
DS1	[1]	1.00e+04	0	5	1
		1.00e+04	0	5	
		1.00e+06	0	5	
		1.00e+06	1	9	
		1.00e+08	5	8	
		1.00e+08	3	5	
DS2	[1]	1.00e+06	0	4	2.625
		1.00e+06	1	5	
		1.00e+08	1	5	
		5.00e+08	3	5	
		2.50e+09	6	6	
		1.00e+10	9	10	
		1.00e+10	9	14	
		1.00e+10	3	5	
		1.00e+10	5	5	
		2.00e+10	2	2	
		2.30e+10	14	19	

Table S1: Datasets used in this study

Parameter ranges for sensitivity analysis

Parameter	Units	Lower bound	Upper bound
C	mg L^{-1}	0.00	0.05
f_r	-	0.00	0.10
$\log_{10}(d)$	-	1.00	4.00
E_{max}*	day^{-1}	612.00	1836.00
EC_{50}*	mg L^{-1}	4.96	14.89
r*	CFU^{-1}	5.33×10^{-9}	1.59×10^{-8}
t_{fs}	days	1.50	2.50

Table S2: Parameter ranges for exponential model. Parameters with * are increased and decreased by 50% of the values used in Fig. 2.

Parameter	Units	Lower bound	Upper bound
C	mg L^{-1}	0.00	0.05
f_r	-	0.00	0.10
$\log_{10}(d)$	-	1.00	4.00
E_{max}*	day^{-1}	612.00	1836.00
EC_{50}*	mg L^{-1}	4.96	14.89
α*	-	0.08	0.24
β*	-	7.07×10^{6}	2.12×10^{7}
t_{fs}	days	2	3

Table S3: Parameter ranges for β-Poisson model. Parameters with * are increased and decreased by 50% of the values used in Fig. 2.

β-Poisson procedure verification

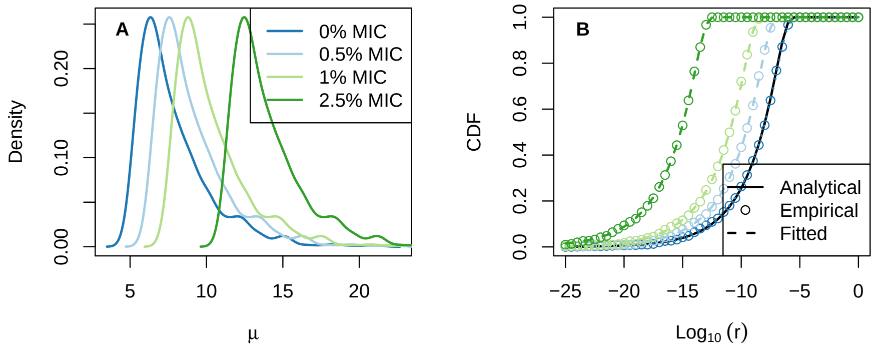

Figure S1: Verify conversion procedure for β-Poisson model. (**A**) Plot of μ at various concentration. (**B**) Comparison of empirical CDF of the sampled r values and the CDF from the fitted Beta distributions. The analytical CDF at 0% MIC is also shown for comparison.

Methods

Using the Simple Death DRM

Suppose we are interested in calculating the response for a pathogen. It is present in an exposure case with $d = 1000$, with 20% of the pathogen being resistant to an antibiotic, and the concentration of antibiotic is $C = 0.025 \times \mathrm{MIC}\,(2\,\mu\mathrm{g\ mL}^{-1}) = 0.05\mu\mathrm{g/mL}$.

- Identify dose-response data for the pathogen. This can be like DS1 or DS2 listed in Table S1.
- Identify t_{fs}. This is the latest time at which some subject shows the first symptom. Suppose $t_{\mathrm{fs}} = 1$ day
- Identify E_{max} and EC_{50} for the antibiotic-pathogen combination of interest. Suppose $E_{\mathrm{max}} = = 1224$ day^{-1}and EC$_{50}$ = 9.93 mg L^{-1}= 9.93 μg mL^{-1}.
- Fit both exponential and β-Poisson models to this dataset and identify the best fitting model, using methods outlined in [2].
- If best fitting model is exponential, go to section titled "Using exponential DRM". If best fitting model is β-Poisson, go to section titled "Using β-Poisson DRM".

Our sensitivity analyses indicate that getting approximate values for t_{fs} are sufficient to predict response. However, it's value is critical to accurately estimate death rate (μ) if using the exponential DRM.

Using exponential DRM

The exponential model is given by:

$$P(d) = 1 - \exp\left(-rd\right)$$

Suppose the best fit for r is given by $\hat{r} = 1.07 \times 10^{-8}$.

- Compute μ by solving

  $$(1 - \exp(-\mu t_{\mathrm{fs}})) = \exp(-\hat{r})$$

  to get

  $$\mu = -\log(1 - \exp(-\hat{r}))/t_{\mathrm{fs}} = 7.97\,\mathrm{day}^{-1}$$

  .

- Compute $\mu_{s,\mathrm{AB}}(C)$ using

  $$\mu_{s,\mathrm{AB}}(C) = \mu + \frac{E_{\mathrm{max}}C}{EC_{50} + C} = 7.97\mathrm{day}^{-1} + \frac{1224\,\mathrm{day}^{-1} \times 0.05\mu\mathrm{g\ mL}^{-1}}{(9.93 + 0.05)\mu\mathrm{g\ mL}^{-1}} = 14.10\,\mathrm{day}^{-1}$$

- Set $\mu_{r,\text{AB}} = \mu = 7.97\,\text{day}^{-1}$
- Compute extinction probabilities for the susceptible and resistant subpopulations using

$$P_{\text{ext},s}(d|f_r, C) = (1 - \exp(-\mu_{s,\text{AB}}(C)t_{\text{fs}}))^{d\times(1-f_r)} \approx 0.999400822$$

and

$$P_{\text{ext},r}(d|f_r, C) = (1 - \exp(-\mu_{r,\text{AB}}t_{\text{fs}}))^{d\times f_r} \approx 0.933317727$$

- Compute total response probability with

$$P(d|f_r, C) = 1 - P_{\text{ext},s}(d|f_r, C)P_{\text{ext},r}(d|f_r, C) \approx 0.067241497$$

- If $\big(1 - P_{\text{ext},s}(d, t|f_r, C)\big)P_{\text{ext},r}(d, t|f_r, C) > (1 - P_{\text{ext},r}(d, t|f_r, C))$, illness is AB treatable. If not, illness is not AB treatable. In this case, this condition evaluates to False and hence the illness is likely not AB treatable.

Using β-Poisson DRM

The β-Poisson DRM is given by

$$P(d) = 1 - \left(1 + \left(\frac{d}{\beta}\right)\right)^{-\alpha}$$

Suppose the best fit parameters are $\hat{\alpha} \approx 0.1615058$ and $\hat{\beta} = 1414958$. Computing response probabilities is more involved and requires access to a function that can fit a beta distribution, such as the `fitdistrplus` package in R ([3]).

- From the values of $\hat{\alpha}$, $\hat{\beta}$, E_{max}, EC_{50} and C, compute α_s and β_s for the susceptible subpopulation. For this, use the algorithm outlined in the Methods section. We get $\alpha_s = 0.1613020$ and $\beta_s = 1.295420 \times 10^{13}$.
- Set $\alpha_r = \hat{\alpha}$ and $\beta_r = \hat{\beta}$ for the resistant subpopulation.
- Compute extinction probabilities for the susceptible and resistant subpopulations using

$$P_{\text{ext},s}(d|f_r, C) = \left(1 + \left(\frac{d \times (1 - f_r)}{\beta_s}\right)\right)^{-\alpha_s} = 1$$

and

$$P_{\text{ext},r}(d|f_r, C) = \left(1 + \left(\frac{d \times f_r}{\beta_r}\right)\right)^{-\alpha_r} \approx 0.999977202$$

- Compute total response probability with

$$P(d|f_r, C) = 1 - P_{\text{ext},s}(d|f_r, C)P_{\text{ext},r}(d|f_r, C) \approx 2.28 \times 10^{-5}$$

- If $\big(1 - P_{\text{ext},s}(d, t|f_r, C)\big)P_{\text{ext},r}(d, t|f_r, C) > (1 - P_{\text{ext},r}(d, t|f_r, C))$, illness is AB treatable. If not, illness is not AB treatable. In this case, this condition evaluates to False and hence the illness is likely AB untreatble.

References

[1] Tacket, C. O. *et al.* Role of EspB in Experimental Human Enteropathogenic Escherichia coli Infection. *Infection and Immunity* **68**, 3689–3695 (2000). URL http://iai.asm.org/cgi/doi/10.1128/IAI.68.6.3689-3695.2000.

[2] Haas, C. N., Rose, J. B. & Gerba, C. P. *Quantitative Microbial Risk Assessment* (John Wiley & Sons, Inc, Hoboken, New Jersey, 2014). URL http://doi.wiley.com/10.1002/9781118910030.

[3] Delignette-Muller, M. & Dutang, C. fitdistrplus: An R Package for Fitting Distributions. *Journal of Statistical Software, Articles* **64**, 1–34 (2015). URL https://www.jstatsoft.org/v064/i04.
